# Body composition changes associated with fasted versus non-fasted aerobic exercise

**DOI:** 10.1186/s12970-014-0054-7

**Published:** 2014-11-18

**Authors:** Brad Jon Schoenfeld, Alan Albert Aragon, Colin D Wilborn, James W Krieger, Gul T Sonmez

**Affiliations:** Department of Health Science, Lehman College, Bronx, NY USA; California State University, Northridge, CA USA; Exercise and Sport Science Department, University of Mary Hardin-Baylor, Belton, TX USA; Weightology, LLC, Redmond, WA USA

## Abstract

It has been hypothesized that performing aerobic exercise after an overnight fast accelerates the loss of body fat. The purpose of this study was to investigate changes in fat mass and fat-free mass following four weeks of volume-equated fasted versus fed aerobic exercise in young women adhering to a hypocaloric diet. Twenty healthy young female volunteers were randomly assigned to 1 of 2 experimental groups: a fasted training (FASTED) group that performed exercise after an overnight fast (n = 10) or a post-prandial training (FED) group that consumed a meal prior to exercise (n = 10). Training consisted of 1 hour of steady-state aerobic exercise performed 3 days per week. Subjects were provided with customized dietary plans designed to induce a caloric deficit. Nutritional counseling was provided throughout the study period to help ensure dietary adherence and self-reported food intake was monitored on a regular basis. A meal replacement shake was provided either immediately prior to exercise for the FED group or immediately following exercise for the FASTED group, with this nutritional provision carried out under the supervision of a research assistant. Both groups showed a significant loss of weight (P = 0.0005) and fat mass (P = 0.02) from baseline, but no significant between-group differences were noted in any outcome measure. These findings indicate that body composition changes associated with aerobic exercise in conjunction with a hypocaloric diet are similar regardless whether or not an individual is fasted prior to training.

## Background

Regimented exercise is considered an important component of a structured weight loss program [1]. Meta-analytic data show that a combined diet-plus-exercise program is more effective in promoting long-term weight loss than a diet-only program [2]. This is consistent with a compelling body of research showing that weight management is predicated on the balance between energy expenditure and intake [3]. Since human movement alters the expenditure side of the energy balance equation, it therefore follows that an increase in physical activity will be accompanied by a reduction in body mass provided energy intake remains constant. Interestingly and importantly, there is evidence in both animal [4] and human [5] models that regular exercise can preferentially reduce abdominal fat, which has been implicated as a primary factor in cardiometabolic risk [6].

The provision of nutrients prior to aerobic exercise has been shown to have a profound impact on the physiological response to the training bout [7,8]. Accordingly, a number of strategies have been devised to take advantage of this phenomenon. One such strategy involves training after an overnight fast to accelerate the loss of body fat [9]. In theory, low glycogen and insulin levels cause the body to shift energy utilization away from carbohydrates, thereby allowing greater mobilization of stored fat for fuel. Findings from several acute studies appear to support this contention, with exercise in the fed state resulting in a reduced entry of long-chain fatty acids in the mitochondria and a corresponding decrease in fat oxidation [10-12]. These results in the fed state have been attributed to an insulin-mediated attenuation of adipose tissue lipolysis, an increased glycolytic flux, and/or a decreased expression of genes involved in fatty acid transport and oxidation [12-14]. There also is evidence that consistent exercise while fasted results in chronic molecular adaptations favorable to fat oxidation. For example, 6 weeks of fasted aerobic training increased the content of intramuscular fatty acid binding protein and uncoupling-protein-3 content to a greater extent than training post-prandially [15]. In addition, regimented fasted training has been shown to promote superior improvements in whole-body glucose tolerance and insulin sensitivity as well as upregulating various lipolytic enzymes compared to exercising while fed [8,16].

Despite an apparent theoretical basis, evidence is scant as to whether fasted aerobic exercise results in greater fat loss over time compared to exercising in the post-prandial state. Although several studies have examined measures of body composition between exercise carried out fasted versus fed [7,8,16], to the authors’ knowledge none have directly investigated this topic during periods of energy restriction. The purpose of this study therefore was to investigate changes in fat mass and fat-free mass following four weeks of volume-equated fasted versus fed aerobic exercise in young women adhering to a hypocaloric diet.

## Methods

### Subjects

Subjects were 20 healthy young female volunteers (age: 22.4 ± 2.8 yrs; height: 163.4 ± 4.7 cms; weight: 62.2 ± 6.5 kgs) recruited from a university population. This sample size was justified by *a priori* power analysis using a target effect size of 0.6, alpha of 0.05 and power of 0.80 with percent body fat as an outcome measure based on previously established results [17]. All participants reported performing aerobic exercise several days a week on a regular basis and several were off-season collegiate track and field athletes. Initial screening required that subjects were between the ages of 18–35, not classified as obese based on a body mass index (BMI; calculated as kg/m^2^) of ≥30, and not involved in a resistance training program at the time of the study. Individuals meeting these criteria were invited to attend a familiarization session where a complete explanation of the study was provided, and a medical history and informed consent were obtained. Participants were excluded from the study if they are found to have any of the following: existing lower body injury; current participation in a resistance training program; metabolic or cardiovascular disorders (including coronary artery disease, cardiac arrhythmias, diabetes, thyroid disease, or hypertension); history of pregnancy within the past 6 months; any condition that would result in stratification as high risk based on criteria set forth by the American College of Sports Medicine [18], and/or taking a prescription or non-prescription weight-loss aid. Those meeting eligibility criteria and willing to participate in the study were scheduled for baseline testing. Approval for the study was obtained from the Institutional Review Board at Lehman College.

### Testing sessions

Testing was carried out in the 24–48 hours prior to beginning the intervention and after the fourth week at completion of the study. For each testing session, subjects reported to the lab in the morning following an overnight fast having refrained from vigorous physical activity, alcohol intake, or consumption of over-the-counter medications for at least 12 hours. Baseline assessments included body mass, height, body composition, and waist circumference, After completion of testing, subjects were pair-matched based on initial body mass measurements and randomly assigned to 1 of 2 experimental groups: a fasted training (FASTED) group that performed exercise after an overnight fast (n = 10) or a post-prandial training (FED) group that consumed a meal prior to exercise (n = 10). The number of athletes and non-athletes were evenly distributed between groups.

### Exercise training intervention

Training consisted of 1 hour of steady-state aerobic exercise performed 3 days per week on a LifeFitness, treadmill (model CLST-0100R-01, Brunswick Corporation, Rosemont, IL) at a 0% grade. Subjects performed a warm-up for the first 5 minutes at an intensity equating to 50% of maximal heart rate (MHR), determined by the formula 220 – age, then increased intensity to 70% MHR for the next 50 minutes, and finished with a 5 minute cool down at 50% MHR. Heart rate monitors (model F7U, Polar Electro Inc, Lake Success, NY) were used to ensure that exercise remained at the appropriate intensity. A low-to-moderate training intensity was used because it has been shown to maximize lipid oxidation during fasted aerobic exercise as compared to higher-training intensities [19]. All training sessions were supervised by research assistants who were upper level undergraduate students in exercise science. Subjects were instructed to refrain from performing any additional structured exercise for the duration of the study.

### Dietary intervention

Subjects were provided with customized dietary plans prepared by one of the researchers (A.A.A.) for the length of the study. In order to facilitate weight loss, energy consumption was set so that subjects remained in a caloric deficit. The determination of energy intake was based on the Mifflin-St. Jeor Equation, which is considered an accurate formula for estimating resting metabolic rate [20]. The formula is as follows:

$$ 10 \times \mathrm{weight}\ \left(\mathrm{kg}\right) + 6.25 \times \mathrm{height}\ \left(\mathrm{cm}\right) - 5 \times \mathrm{age}\ \left(\mathrm{y}\right) - 161 $$

The formula was multiplied by a moderate activity factor (1.5) to estimate energy balance, and the total was then reduced by 500 calories to impose a caloric deficit. Dietary protein intake was set at 1.8 g/kg of body mass, as higher protein consumption has been shown to help offset losses in lean tissue mass and promote greater adherence to the nutritional regimen [21,22]. After accounting for protein intake, dietary fat was 25-30% of total calories and the remaining calories were obtained from carbohydrate. Sample meal plans were provided to guide the participants in acceptable food choices.

Dietary plans included provision of a meal replacement shake (Pursuit Recovery, Dymatize Nutrition, Dallas, TX). The shake contained 250 calories consisting of 40 g carbohydrate, 20 g protein, and 0.5 g fat. On exercise days, FED consumed the shake immediately prior to the exercise bout and FASTED consumed the shake immediately after finishing the bout. Shakes were consumed under the supervision of a research assistant to ensure adherence within the context of the subject’s respective participation in the fed or fasted protocol.

Dietary adherence was assessed by self-reported food records using MyFitnessPal.com (http://www.myfitnesspal.com), which were collected and analyzed during on a daily basis to ensure that intake was not based on recall. Subjects were instructed on how to properly record all food items and their respective portion sizes that were consumed for the designated period of interest. Each item of food was individually entered into the program, and the program provided relevant information as to total energy consumption, as well as amount of energy derived from proteins, fats, and carbohydrates over the length of the study. Continued nutritional guidance was provided to the subjects at the time of each training session by the research team to encourage dietary adherence.

### Anthropometrics and body composition measurements

Height and body mass measurements were made using a double beam scale. Circumference measurements of the waist was made using an Intelametrix tape measure (Intelametrix Inc., Livermore, CA) according to established criteria [18]. Body mass index (BMI) was calculated as body mass in kg divided by height in meters squared. Percent fat mass and lean body mass was obtained via air displacement plethysmography (ADP) using the BodPod body composition analyzer (model 2000a, Life Measurement, Concord, CA) as per the user manual and described previously in the literature [23]. ADP has been shown to have good validity in measuring body fat percentage when compared to dual x-ray absorptiometry in the sampled population [24,25]. Subjects were tested in tight clothing (either compression shorts and a sports bra or a swimsuit) and Lycra swim cap. Based on body mass and volume as well as through body density, total fat mass, total fat free mass and body fat percentage were calculated by the BodPod system software.

### Statistical analyses

Normality assumptions were checked using a one-sample Kolmogorov-Smirnov test; all data was found to meet normality assumptions. Independent t-tests were used to assess differences in baseline measurements between groups as well as energy and macronutrient intake over the length of the study period. Cohen’s D effect sizes were calculated for all pre- to post-study outcome measures using the following formula:

$$ {M}_1-{M}_2/SD $$

where *M*_*1*_ represents the pre-study mean, *M*_*2*_ represents the post study mean, and; *SD* represents the pooled standard deviation.

All other data was modeled using a linear mixed model estimated by a restricted maximum likelihood algorithm. Treatment was included as the between-subject factor, time was included as the repeated within-subjects factor, and time x treatment was included as the interaction. Repeated covariance structures were specified as unstructured. All analyses were performed using S-Plus 8.2 (Tibco Spotfire, Boston, MA). Effects were considered significant at P ≤0.05, and trends were declared at 0.05 < P ≤0.10. Data are reported as $\overline{x}$ ± SD.

## Results

**Figure 1:**
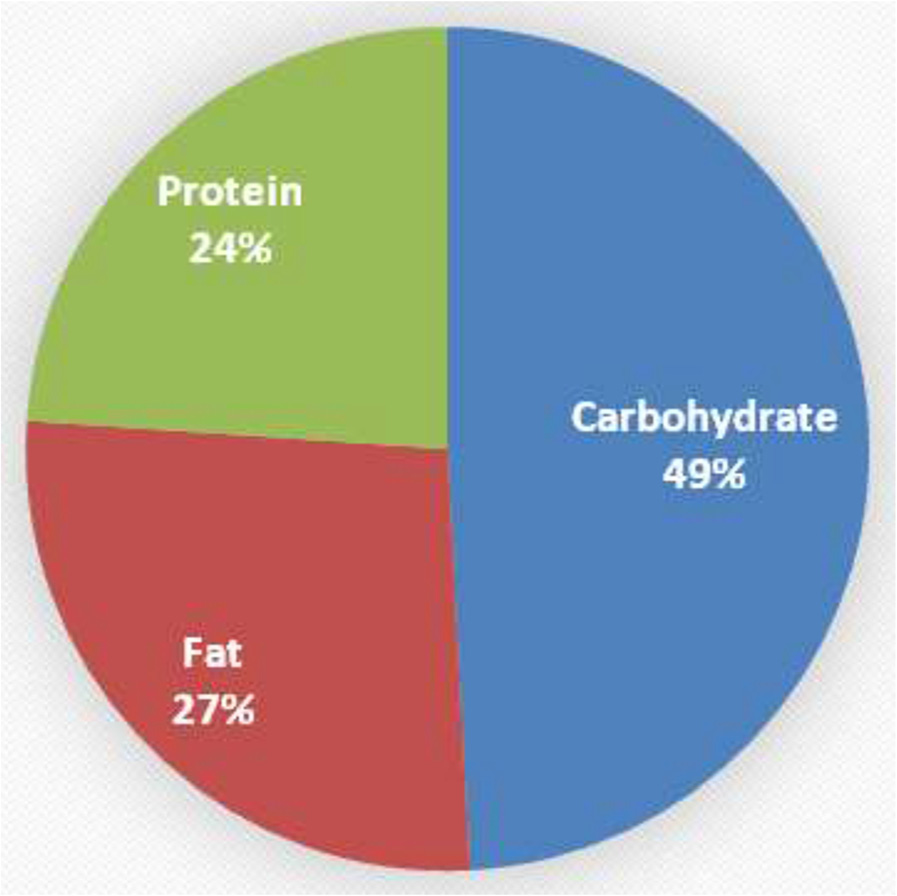
**Percent macronutrient intake for FASTED.**

**Figure 2:**
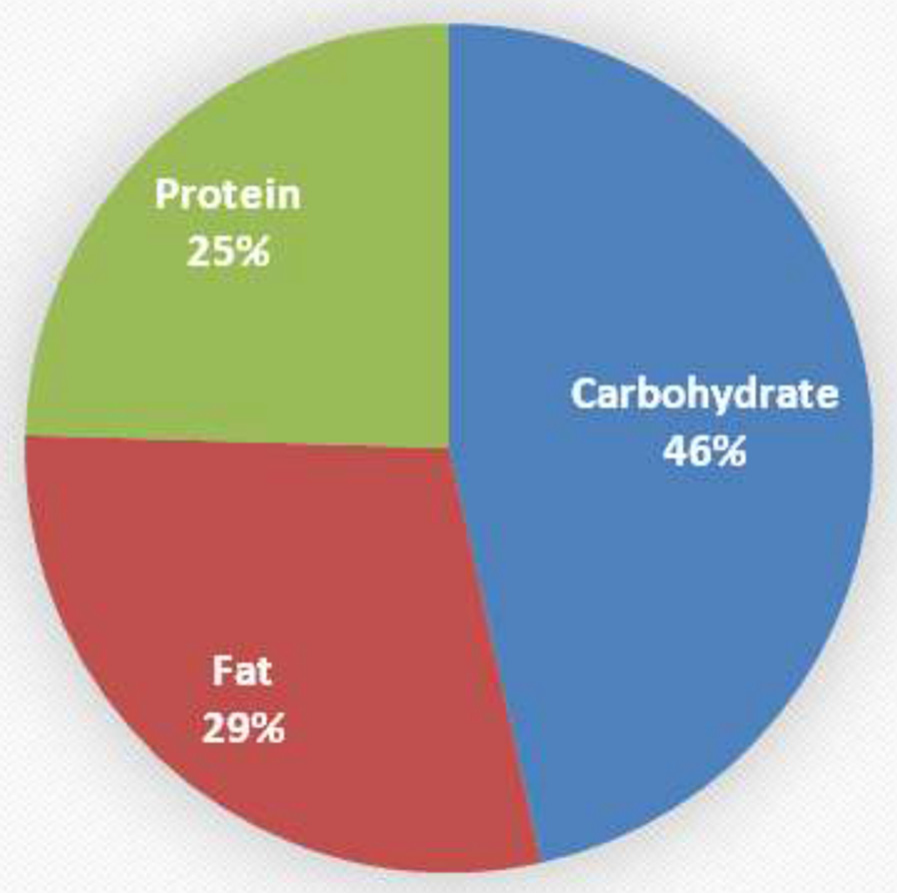
**Percent macronutrient intake for FED.**

The FED group was significantly younger than the FASTED group (21 ± 1.7 yrs versus, 23.8 ± 3.0 yrs, respectively; p = 0.02). No other significant group differences were noted in any baseline measure. Total energy and macronutrient consumption was not different between groups over the length of the study period (see Table 1). FASTED reported consuming 1236 calories/day; FED reported consuming 1277 calories/day. The reported nutritional consumption for both groups was below that prescribed in the individual meal plans. Figures 1 and 2 display macronutrient percentage intake in FASTED and FED, respectively.

**Table 1:** **Nutritional intake between groups**

**Group**	**Calories**	**Carbohydrate (g)**	**Fat (g)**	**Protein (g)**
FASTED	1236 ± 177	152 ± 38	37 ± 7	74 ± 15
FED	1277 ± 137	148 ± 19	41 ± 13	78 ± 21

Pre- to post-study results for each outcome measure are presented below. Table 2 summarizes these findings.

**Table 2:** **Pre- vs. Post-study body composition measures**

**MEASURE**	**FASTED-PRE**	**FASTED-POST**	**ES**	**FED-PRE**	**FED-POST**	**ES**
Weight (kg)	62.4 ± 7.8	60.8 ± 7.8*	0.21	62.0 ± 5.5	61.0 ± 5.7*	0.18
BMI	23.4 ± 2.9	22.8 ± 3.0*	0.20	23.3 ± 2.5	22.9 ± 2.5*	0.16
Body Fat%	26.3 ± 7.9	25.0 ± 7.7	0.17	24.8 ± 8.4	24.1 ± 8.5	0.08
Waist (cm)	77.5 ± 6.4	75.9 ± 6.9	0.24	77.7 ± 9.4	75.7 ± 8.6	0.22
Fat Mass (kg)	16.5 ± 5.5	15.4 ± 5.5*	0.20	15.7 ± 6.3	15.0 ± 6.1*	0.11
Fat Free Mass (kg)	45.9 ± 6.7	45.4 ± 6.1	0.08	46.3 ± 3.8	46.1 ± 4.3	0.05

An asterisk* indicates a significant effect from baseline values.

### Body mass

There was no significant interaction between time and group, and there was no significant effect of group. There was a significant effect of time (P = 0.0005), with a decrease in body mass from pre- to post.

### BMI

There was no significant interaction between time and group and there was no significant effect of group. There was a significant effect of time (P = 0.0005), with a decrease in BMI from pre- to post.

### Percent body fat

There was no significant interaction between time and group, and there was no significant effect of group. There was a trend for an effect of time (P = 0.06), with a trend for a decrease in percent body fat from pre- to post.

### Waist circumference

There was no significant interaction between time and group, and no significant effect of group. There was a trend for an effect of time (P = 0.07), with a trend for a decrease in waist circumference from pre- to post.

### Fat mass

There was no significant interaction between time and group, and no significant effect of group (P = 0.88). There was a significant effect of time (P = 0.02), with a decrease in fat mass from pre- to post.

### Fat-free mass

There was no significant interaction between time and group, and no significant effects of group or time.

## Discussion

To the authors’ knowledge, this is the first study to investigate body composition changes associated with aerobic exercise performed in the fasted versus fed state while subjects maintained a caloric deficit. It has been hypothesized that exercising when fasted forces the body to rely on using fat as a substrate rather than carbohydrate, thereby reducing body fat to a greater extent than performance of post-prandial exercise. Our results refute the veracity of this hypothesis. Although both groups lost a significant amount of weight and fat mass, no differences were seen between conditions in any outcome measure regardless of pre-exercise feeding status.

Van Proeyen et al. [16] investigated the effects of fasted versus fed aerobic exercise on metabolic parameters and body composition in a convenience sample of physically active young men while maintaining an energy-matched hyper-caloric diet (∼ + 30% kcal day^−1^). Subjects performed a combination of moderately intense cycling and running exercise for 60–90 minutes, 4 days per week. After 6 weeks, the fed group significantly increased body mass by 1.4 kg while no significant increases were found in the fasted group, suggesting an attenuation of weight gain from fasted training. Follow-up work from the same lab employing the same basic training protocol but with subjects consuming an isocaloric diet (based on 4-day dietary analysis) showed no differences in body weight change between fasted versus fed conditions [8]. Recently, Gillen et al. [7] compared body composition changes associated with exercising in the fasted versus fed state in 16 overweight/obese women. Exercise consisted of high-intensity interval training (ten 60-second cycling intervals at 90% maximal heart rate with a 1:1 work/recovery ratio) performed 3 days per week for 6 weeks. Subjects were instructed to maintain their pre-intervention eating patterns throughout the study period. At study’s end, body mass remained unchanged from baseline but lower body fat was noted both in the abdominal and leg regions as well as the whole body level in both groups. No significant differences were found between conditions.

Our results are novel in that subjects consumed a supervised hypocaloric diet throughout the intervention period. Loss of body mass is predicated on shifting energy balance to favor expenditure over intake [3]. This is consistent with the First Law of Thermodynamics, which essentially states that energy is neither created nor destroyed, but rather changed from one form to another. Thus, our approach allowed for a controlled investigation of the effects of fasted exercise on body composition under conditions favorable for fat loss. Moreover, to optimize the proposed benefits of training while fasted, exercise was performed at low-to-moderate intensities. This is consistent with acute research showing that lipolysis is blunted during performance of higher- [14,26] but not lower-intensity exercise when carried out in the fed state [10,11,27]. Despite these accommodations, fasted exercise showed no beneficial effects compared to training post-prandially.

The theoretical basis behind a fat-burning advantage to fasted exercise is predicated on increasing lipid oxidation during training bout. However, this ignores the dynamic nature of the human body, which continually adjusts its use of substrate for fuel. There is evidence that a greater utilization of fat for fuel during a given time period is compensated by a greater carbohydrate utilization later in the day [28]. Hence, fat burning must be considered over the course of days — not on an hour to hour basis — to meaningfully assess its impact on body composition [29]. In support of this contention, Paoli et al. [30] compared differences in 24-hour fat metabolism associated with performance of moderate-intensity cardiovascular treadmill exercise in the fasted versus fed state. Food quantity and quality was identical between conditions over the ensuing 24-hour recovery period. Consumption of breakfast for the fed condition resulted in a significant increase in respiratory exchange ratio (RER) compared to fasting (0.96 vs. 0.84, respectively). However, at 12 hours post-exercise RER was significantly lower in the fed versus fasting condition and the difference remained significant after 24 hours.

Any potential increases in fat oxidation from fasted exercise might be neutralized by an increase in the thermic effect of exercise from eating pre-exercise. Lee et al. [31] compared the acute thermogenic effects of an exercise bout performed in either a fasted state or following ingestion of a glucose/milk (GM) beverage. Employing a within-subject design, 10 male college students performed four experimental conditions in randomized order: low intensity, long duration exercise with GM; low intensity, long duration exercise without GM; high intensity, short duration exercise with GM, and; high intensity, short duration exercise without GM. Results showed that consumption of the GM beverage increased excess post-exercise oxygen consumption to a significantly greater extent than exercise performed while fasted in both high and low intensity conditions. Similar findings have been reported in other controlled trials [32,33].

The study had several notable limitations that must be taken into account when attempting to draw evidence-based conclusions. First, the duration of the testing period was fairly short, lasting just four weeks. While this period is certainly sufficient to attain significant fat loss, it remains possible that subtle changes between protocols would take more time to manifest. Second, although every attempt was made to control the subject’s nutritional intake, it remains possible that differences in energy and/or macronutrient intake may have confounded results. There is evidence that self-reported consumption of food can vary by as much as 18% even after being trained by dieticians [34]. The fact that food diaries were collected daily provides confidence that a lapse in memory did not influence what was reported, but the inability to accurately measure portions and/or intent to deliberately misreport intake cannot be ruled out. Given that mean weight loss across groups was somewhat less than anticipated, it seems reasonable to assume that subjects did in fact underreport the amount of calories consumed, which would explain the attenuated results. Similarly, although subjects were instructed not to partake in any other structured exercise other than activities of daily living, there is no way to assure that they adhered to this request. Third, the use of young women as participants raises the possibility of confounding by menstrual cycles. The fact that testing was carried out exactly one month apart provides some degree of confidence that this did not influence results. However, it is not uncommon for women to have irregular menses, which may have affected hydration status and thus altered body composition measures. Fourth, the FASTED subjects consumed nutrients immediately following training; it is not clear what, if any, impact would be seen on results by delaying consumption for longer periods post-exercise. Finally, results are specific to young, non-obese women and cannot necessarily be generalized to other populations. For example, employing fasted-state, low- to moderate-intensity aerobic exercise is a popular tactic in physique sports where pushing extremes of leanness is one of the primary aims of contest preparation [35]. The applicability of the present study’s results to such endeavors remains open to question.

## Conclusion

In conclusion, our findings indicate that body composition changes associated with aerobic exercise in conjunction with a hypocaloric diet are similar regardless whether or not an individual is fasted prior to training. Hence, those seeking to lose body fat conceivably can choose to train either before or after eating based on preference. It should be noted that given the small sample size and short study duration, we cannot rule out the possibility that either condition might confer a small benefit over the other with respect to fat loss. Further study is warranted in a longer term trial with a greater number of participants.
